# Veterinary perspectives on the urbanization of leishmaniosis in Morocco

**DOI:** 10.1186/s13071-024-06411-5

**Published:** 2024-08-19

**Authors:** Clara M. Lima, Maria Bourquia, Abderrahmane Zahri, Nada Haissen, Nuno Santarém, Luís Cardoso, Anabela Cordeiro da Silva

**Affiliations:** 1 Host-Parasite Interaction Group, i3S, Institute for Research and Innovation in Health, University of Porto, Porto, Portugal; 2 Microbiology Laboratory, Department of Biological Sciences, Faculty of Pharmacy, University of Porto, Porto, Portugal; 3 Unité de Parasitologie et Maladies Parasitaires, Département de Pathologie et Santé Publique Vétérinaires, Institut Agronomique et Vétérinaire Hassan II, Rabat, Morocco; 4 Department of Veterinary Sciences, and Animal and Veterinary Research Centre (CECAV), University of Trás-os-Montes e Alto Douro, Vila Real, Portugal; 5 Associate Laboratory for Animal and Veterinary Sciences (AL4AnimalS), Vila Real, Portugal

**Keywords:** ELISA, Leishmaniosis, LicTXNPx, Morocco, One Health, PCR, rKDDR-ELISA, SPLA-ELISA, Survey, Veterinarians

## Abstract

**Background:**

Leishmaniosis caused by *Leishmania infantum*, *L. major* and *L. tropica* is endemic in Morocco. Growing evidence of both human and canine *Leishmania* infections in urban centres has been reported. Since many forms of the disease are zoonotic, veterinarians play an important role in leishmaniosis control by intervening at the parasite host level. This study aimed to bring together One Health principles to connect canine and feline leishmaniosis epidemiology within urban centres of Morocco (Rabat and Fez) and assess the level of awareness of Moroccan veterinarians about facing this threat.

**Methods:**

A molecular survey was conducted for *Leishmania* DNA detection in canine (*n =* 155) and feline (*n* = 32) whole-blood samples. Three conventional polymerase chain reaction (PCR) protocols were implemented. The first PCR aimed at identifying infected animals by targeting *Leishmania* spp. kinetoplast minicircle DNA (kDNA). The second and third PCR targeted the *Leishmania* internal transcribed spacer region (ITS-1) and the *Leishmania* small subunit ribosomal RNA (SSUrRNA) gene, respectively, aiming at identification of the infecting species after Sanger sequencing-positive amplicons. Total immunoglobulin G (IgG) against *Leishmania* spp. was evaluated in 125 dogs by enzyme-linked immunosorbent assays (ELISA) using an in-house protocol, including three *Leishmania*-specific antigens (SPLA, rKDDR and LicTXNPx). Sera from 25 cats were screened for total IgG to *Leishmania* spp. by an indirect immunofluorescence antibody test (IFAT). An online questionnaire was presented to Moroccan veterinarians addressing their knowledge and practices towards animal leishmaniosis.

**Results:**

Overall, 19.4% of the dogs tested positive for *Leishmania* kDNA and ITS-1 and sequencing revealed infection with *L. infantum* among PCR-positive dogs. These animals presented a wide range of ELISA seropositivity results (16.7%, 34.9% and 51.6%) according to the tested antigens (rKDDR, SPLA and LicTXNPx, respectively). Use of kDNA-PCR revealed 12.5% cats positive to *Leishmania* spp. otherwise found to be seronegative by IFAT.

**Conclusions:**

A considerable prevalence of infection was identified in dogs from urban centres of Morocco. Additionally, this is the first report of feline infection with *Leishmania* spp. in this country and in urban settings. Moroccan veterinarians are aware that animal leishmaniosis is endemic in Morocco, representing a public health threat, and are knowledgeable about canine leishmaniosis diagnosis and treatment.

**Graphical Abstract:**

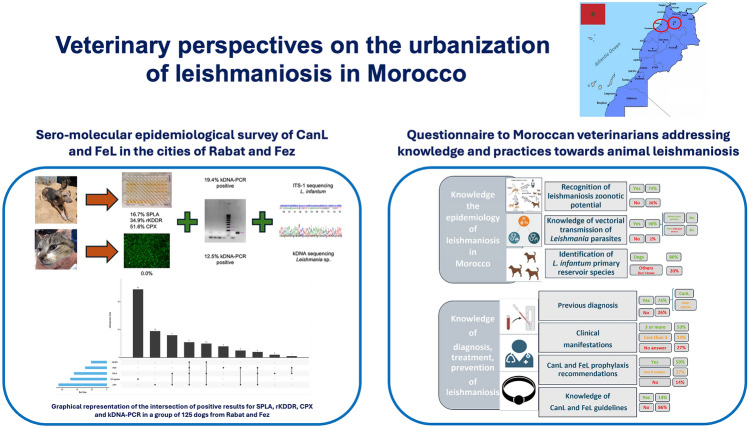

**Supplementary Information:**

The online version contains supplementary material available at 10.1186/s13071-024-06411-5.

## Background

*Leishmania* parasites have infected vertebrates since ancient times [1]. Among the more than 50 known *Leishmania* spp., which are mainly transmitted by blood-feeding female phlebotomine sand flies, around 20 are known to infect humans and around 12 to infect dogs. Diseases caused by *Leishmania* spp. are endemic in more than 90 countries. They are far from being eradicated and contribute largely to Neglected Tropical Disease (NTD)-associated mortality [2]. Zoonotic visceral leishmaniosis (VL) is endemic in the Maghreb region, as is zoonotic and anthroponotic cutaneous leishmaniosis (CL) [3–5]. In this epidemiological context, zoonotic VL is caused by *Leishmania infantum*, whereas *Leishmania major* and *Leishmania tropica* are aetiological agents of CL. In Morocco, the burden of CL is higher than that of VL [6–8]. Between 1997 and 2018, a total of 78,001 CL (with 54% assumed to be caused by *L. major* and 43% by *L. tropica*) and 2298 VL cases were reported [5, 7, 8]. Sporadic *L. infantum*-associated cutaneous lesions accounted for 3% of new diagnoses of CL [7, 8]. While VL is a chronic wasting pathology with a fatal outcome if untreated, the cutaneous presentations are not life-threatening. Nonetheless, they are long lasting and lead to social discrimination and stigmatization [9, 10].

Overall, *L. infantum* and *L. tropica* are endemic in the northern regions of Morocco, with *L. tropica* being also endemic in the central and western semi-arid areas [5, 11, 12]. As for *L. major*, it has been found in the south of the country (in the Saharan region). These differences can be attributed to the dispersal of *Leishmania* vectors, mostly dictated by latitude, altitude, landscape, environment and climate [13, 14].

Domestic dogs are the primary urban reservoirs of *L. infantum* [15] and canine leishmaniosis (CanL) is an impacting zoonotic parasitic vector-borne disease. In Morocco, CanL is caused by zoonotic *L. infantum*, and between 2001 and 2021 the overall national seroprevalence was estimated at 24% [11]. This is 10% higher than the estimated value for the period of 1982 to 2001 [15]. Therefore, CanL surveillance and control are of the utmost importance for public health. Most of these surveillance efforts are carried out by veterinarians and understanding their degree of preparedness is essential. Moreover, leishmaniosis is considered a public health priority in Morocco, notifiable since 1995. Furthermore, human leishmaniosis is considered a rural and peri-urban disease, affecting mostly children and those living in low socioeconomic conditions, where the proximity between humans and animals favours zoonotic transmission [5, 6, 16]. Following this and considering One Heath responsibilities and the World Health Organization (WHO) recommendations for top key interventions to overcome NTD, veterinarians' role in effective disease control is essential [2]. Zoonotic VL [16] and CL [12] case detection is increasing in urban settings. Thus, in this context, it is important to generate data that evaluate whether surveillance should be extended beyond the traditional rural context. Besides, given the growing debate over the role of cats in *L. infantum* transmission, followed by evidence of a high prevalence of feline infection and the infectiousness of cats to phlebotomine sand flies [17–19], both canine and feline species should be monitored when considering zoonotic leishmaniosis surveillance.

In epidemiological settings where zoonotic *Leishmania* spp. occurs in sympatry among vectors, animal hosts and humans, field and laboratory surveillances are the foundation for monitoring infection and disease burden and early recognition of epidemics [20, 21]. Laboratory diagnosis is challenged by the outcome of complex and effective parasite interaction with the host. This is reflected in a spectrum of presentations, ranging from subclinical infection to severe disease, which may not always be accompanied by the development of detectable humoral responses [22]. The magnitude and specificity of both humoral and cellular responses hamper the use of a single diagnostic approach to detect *Leishmania* infections [23–25]. The combination of both molecular and multiparameter serological approaches to investigate infection improves clinical CanL diagnosis [23, 25, 26], providing remarkable advantages in the identification of subclinical infections [27–29]. Serological surveys of leishmaniosis largely rely on crude *Leishmania* promastigote soluble antigens. Previous reports have highlighted the need to critically evaluate *Leishmania*-specific seroreactivity against such crude antigens as distinct patterns of seroreactivity can be found even in non-endemic regions because of a polyclonal or cross-seroreactivity to other pathogens [26]. Therefore, application of multiparameter tools for leishmaniosis diagnosis and epidemiological studies is justified.

Based on these premises, this study aimed to assess Moroccan veterinarians’ knowledge on animal leishmaniosis management and provide updated epidemiological data on canine and feline infection with *Leishmania* spp. in the cities of Rabat and Fez. Rabat is the capital city of Morocco, located along the Atlantic Ocean coastline. It is the seventh largest city in the country. Fez is the second largest city in Morocco and the capital of the Fès-Meknès region, located in the northern interior of Morocco (northwest of the Atlas Mountains). To achieve these goals, we explored the potential of molecular and multiparameter serological approaches for detecting animal leishmaniosis while investigating how veterinarians across the country perceive this disease and which practices are most applied for its management.

## Methods

### Study area

The study was conducted between December 2022 and December 2023 in two urban settings of northern Morocco, the cities of Rabat and Fez. Inclusion criteria included previous reports on the endemicity of leishmaniosis in the region, but not within urban centres [11], and the existence of at least one animal shelter and a veterinary practice in town.

Canine and feline sampling sites included an animal shelter in Rabat city, an animal shelter in Fez, a clinical practice located in Rabat (“Institut Agronomique et Vétérinaire” [IAV] Hassan II Veterinary Teaching Hospital) and a private veterinary practice in Fez. Collaborating institutions were selected based on the willingness to participate in the research after being contacted with a description of the scope of the study and a request to collaborate. Samples were collected or obtained from convenience sampling of randomly selected animals living in shelters and others admitted for a consultation at collaborating veterinary centers. Feline samples were also conveniently collected from cats admitted for a consultation or to trap-neuter-release (TNR) programmes at IAV Hassan II Veterinary Teaching Hospital.

### Clinical data and blood collection

Clinical manifestations compatible with leishmaniosis were registered for dogs [30] and cats [18, 30–32] along with the city, animal's sex, estimated age, breed, housing/habitat, antiparasitic prophylaxis and antiviral vaccination (whenever possible). No information on antiparasitic prophylaxis and antiviral vaccination was available for stray cats. Whenever two or more clinical signs or manifestations compatible with leishmaniosis were observed, the animal was described as “CanL-suspect” or “feline leishmaniosis (FeL)-suspect”. Peripheral blood samples were obtained after oral informed consent from the animal owner or legal representative. Samples were transported to the IAV Hassan II Parasitology and Parasitic Diseases Unit, where they were aliquoted and stored at –20 °C until testing.

An ethical clearance statement for this study was issued by IAV Hassan II “Comité d'Ethique en Sciences et Santé Animales et Santé Publique Vétérinaire” (CESASPV).

### Molecular survey

Genomic DNA was extracted from 200 μl of whole blood (in EDTA) collected from 155 dogs and 32 cats. Extraction was performed by a commercial kit (DNAeasy Blood and Tissue Kit, Qiagen, Hilden, Germany) according to the manufacturer’s instructions. Individual DNA aliquots were frozen at –20 °C until further processing. Three polymerase chain reaction (PCR) protocols were implemented aiming at *Leishmania* detection and further species identification by sequencing and assembling the positive products. First, for *Leishmania* spp. detection, a kinetoplast minicircle DNA (kDNA)-PCR was performed on all samples. The PCR used primers RV1 (forward) (5′CTTTTCTGGTCCCGCGGGTAGG) and RV2 (reverse) (5′ CCACCTGGCCTATTTTAC ACCA), targeting a conserved region of *Leishmania* sp. kDNA, through amplification of a ∼ 145 bp product [33]. Then, all kDNA-PCR positive samples were subject to two new PCR protocols, targeting different regions of the small subunit ribosomal ribonucleic acid gene. These included a conventional PCR protocol to detect *Leishmania* internal transcribed spacer 1 (ITS-1) region of the *Leishmania* small subunit ribosomal RNA gene (SSU-rRNA), using LiTSR (forward) (5′-CTGGATCATTTTCCGATG-3′) and L5.8S (reverse) (5′-TGATACCACTTATCGCACTT-3′) primers [34], and a *Leishmania*-specific nested PCR (LnPCR) targeting *Leishmania* SSUrRNA gene, using the set of primers R221 (GGTTCCTTT CCTGATTTACG) and R332 (GGCCGGTAAAGGCCGAATAG) for the first amplification, followed by reamplification of the previous products using primers R223 (TCCCATCGCAACCTCGGTT) and R333 (AAAGCGGGCGCGGTGCTG) [35]. Thermocycling conditions are described in Additional file 1: Table S1.

PCR products were submitted to electrophoresis for 60 min, 120 V, in ultrapure agarose gel (2%) (Ultra-pure grade agarose, NZYtech, Lisbon, Portugal) prepared in 1 × Tris-acetate-EDTA buffer (TAE) supplemented with 0.2 μg/ml nucleic acid stain (GreenSafe Premium, NZYtech, Lisbon, Portugal). Qualitative identification of PCR products was performed by gel visualization under UV light (530 nm) (ChemiDoc XRS + system; Bio-Rad, Hercules, CA, USA) and complemented with quantitative analysis by Image Lab software version 6.1, Lane profile tool (Bio-Rad, Inc). Genomic DNA from a *L. infantum* laboratory strain (MHOM/MA/67/ITMAP-263) was used as positive control. Contaminations were assessed by lack of amplification on a blank sample consisting of water instead of DNA. Results were compared to a 100-bp molecular marker (Ladder V, NZYTech).

Whenever required for sequencing, PCR products were retrieved from the electrophoresis gel, purified and quantified. The agarose gel extracted DNA products were purified using NucleoSpin Gel and PCR Clean-up columns (Macherey-Nagel GmbH&Co., Düren, Germany) and quantified by nanodrop (ND1000 spectrophotometer, NanoDrop).

Positive ITS-1 and SSUrRNA amplicons and kDNA-positive cats were Sanger sequenced in both directions, using the same forward and reverse primers (AB Applied Biosystems, Thermo Fisher Scientific, Waltham, MA, USA). Nucleotide (nt) sequences were assembled and compared for similarity with other sequences available at the National Center for Biotechnology Information (NCBI) GenBank using the Basic Local Alignment Search Tool (BLAST; http://blast.ncbi.nlm.nih.gov/Blast.cgi).

### Serological survey

Canine (*n* = 125) sera were tested for anti-*Leishmania* total immunoglobulin G (IgG) using an in-house enzyme-linked immunosorbent assay (ELISA). The canine ELISA protocols were based on three soluble *Leishmania*-specific antigens, including soluble promastigote *Leishmania* crude proteins (SPLA, 10 μg ml^−1^)l recombinant *Leishmania* kinesin degenerated derived repeats (rKDDR, 5 μg ml^−1^) and *L. infantum* cytosolic tryparedoxin peroxidase (LicTXNPx, 5 μg ml^−1^), as previously described [23, 26, 29]. Canine serum samples were tested at 1:1500 dilution (100 μl/well) using 100 μl/well of rabbit-produced anti-dog IgG antibody conjugated with horseradish peroxidase (Sigma, St. Louis, MO, USA) diluted to 1:1500 in PBS-Tween 0.05%. Sera from a dog naturally infected with *L. infantum* was used as a CanL-positive control. All samples were tested in technical triplicates for each antigen. The blank for each antigen including a conjugated control, containing no sera, was also tested in triplicate for each antigen. At least two independent assays were performed. Optical densities (ODs) were recorded at 490 nm for every single measurement using an automated ELISA plate reader (Synergy 2, BioTek Instruments, Winooski, VT, USA). Seropositivity for CanL was determined by cut-offs previously calculated for this ELISA protocol by means of receiver operating characteristic (ROC)-curve analysis [23], including 0.075 OD for SPLA, 0.098 OD for rKDDR and 0.04 OD for LicTXNPx.

Feline (*n* = 25) seropositivity for anti-*Leishmania* total IgG was evaluated by indirect immunofluorescence antibody test (IFAT). The previously validated FeL-IFAT [36] was implemented and reproduced with minor modifications, as previously described [37]. Positivity was considered whenever > 50% of the acetone fixed *L. infantum* promastigotes produced clear green fluorescence on cytoplasm, cellular membranes and flagella. Serum from a *L. infantum* naturally infected cat was used as a positive control. The negative control or blank consisted of a conjugated control, containing no sera. Positivity cut-off was defined at 1:80 serum dilution.

### Questionnaire to veterinarians

An anonymous and self-administered questionnaire directed to Moroccan veterinarians was structured on Google Forms and disseminated online to collect information on veterinarians' knowledge and practices regarding animal leishmaniosis in Morocco. The questionnaire was constructed in English and later translated into French by a bilingual researcher from IAV Hassan II, Rabat, Morocco. Before dissemination, the questionnaire was tested and evaluated by five non-Moroccan and three Moroccan veterinarians to assess its conformity. The time for completion was estimated at 15 min. The questionnaire was divided into two parts. The first part aimed at a demographic characterization, including a description of the participants gender, age, graduating institution, clinical experience (years in practice, type of practice) and geographical location. The second part focused on veterinarians’ knowledge of leishmaniosis epidemiology in Morocco and explored clinicians’ experience with leishmaniosis prevention, diagnosis and treatment. In this section, veterinarians were asked to identify leishmaniosis as a zoonotic disease, identify animal reservoirs and transmission vectors, recognize the most prevalent *Leishmania* sp./spp. in Morocco responsible for human and animal disease and describe clinical manifestations associated with previously diagnosed cases of leishmaniosis (independently of the animal species), treatments applied and their position regarding the recommendation of prophylactic measures against animal leishmaniosis. Answers could be selected from multiple choices (Additional file 2: Figure S1).

The questionnaire was disseminated between December 2022 and June 2023 among approximately 220 potential respondents, taking advantage of veterinary-dedicated social media platforms in Morocco and electronic e-mail addresses used in a previous questionnaire organized by the IAV “Unité de Parasitologie et Maladies Parasitaires, Département de Pathologie et Santé Publique Vétérinaires, Rabat, Morocco”. Information on the scope and purpose of the questionnaire was made available upfront. Eligibility criteria to participate in the survey included a graduation in veterinary medicine, being currently active in clinical practice and having access to an internet-connected device. Participation was voluntary and anonymous.

### Data analysis

Statistical analysis was performed using IBM SPSS Statistics for Window, version 27.0 (IBM Corp, Armonk, NY, USA) and Microsoft Excel (Microsoft Corp., Redmond, WA, USA). Pearson Chi-square test (*χ*^2^) and Fisher’s exact test (FET) were used to compare percentages; the McNemar test compared percentages obtained from paired samples (i.e. from the same animal); the Cohen’s kappa coefficient assessed agreement beyond chance; 95% confidence intervals were defined by the exact binomial test. To allow a bivariate statistical analysis of the questionnaire results, the following variables were regrouped: age, years in practice, type of practice and practice location. Statistically significant differences were considered whenever the *p* (probability) value was < 0.05.

## Results

### Demographic, clinical and prophylactic characterization of the studied animals

Canine whole blood and serum were available from 155 and 125 sampled dogs, respectively. Feline whole blood and serum were available from 32 and 25 cats, respectively. The canine group comprised 81 dogs from the city of Rabat (52 dogs from a local shelter and 29 owned) and 75 dogs from the city of Fez (46 dogs from a local shelter and 29 owned). Most of the dogs (92.3%; *n* = 144/156) were adults (age 3–9 years). Besides, two dogs (2/156) were puppies (age < 1 year), eight dogs (8/156) were young adults (1 to 3 years), and two (*n* = 2/156) were seniors (age > 12 years). Regarding breed, most were mongrels (100/156) followed by crossbreeds (43/156) and pure breeds (13/156) (Additional file 3: Table S2).

The feline group included 27 (81.8%) stray and 6 (18.2%) owned cats (5 living strictly indoor and 1 with outdoor access) (Additional file 4: Table S3). All stray cats had been admitted at IAV Hassan II Veterinary Teaching Hospital for TNR. Owned cats had been admitted at this same institution following the need for a veterinary appointment. Male cats (8/31) were either neutered (*n* = 6) or intact (*n *= 2), while female cats were all intact (Additional file 4: Table S3).

Clinical findings from the animal's physical examination and clinical history were registered for 156 dogs and 31 cats. From all dogs, 54 (34.6%) were found ill, among which 48 (30.8%) were considered CanL suspect following the identification of at least two clinical manifestations compatible with the disease (Additional file 5: Table S4). The most identified clinical manifestations of CanL included cutaneous lesions (onychogryphosis, 70.8%; generalized alopecia, 31.23%; crusts, 20.4%; pinna lesions or crusts, 4.2%; generalized hyperkeratosis and/or hyperkeratosis of the elbows, 2.1%; pyoderma, 2.1%; seborrhea, 2.1%; skin nodules hyperpigmentation, 2.1%), ocular lesions (conjunctivitis, 2.1%; peri-ocular alopecia, 2.1%) and mucocutaneous lesions (nasal planum hyperkeratosis, 10.41%). Besides, other more general clinical findings, including lymphadenopathy (54.2%), weight loss (20.8%) and cachexia (12.5%), were also found (Additional file 5: Table S4).

All sheltered dogs were overdue for internal and external parasite prophylaxis and vaccines. In the group of owned dogs, 50% were up to date with rabies and core vaccination. Canine intestinal parasite preventives were administered monthly to 33.3% of the dogs, quarterly to 1.8% of the dogs and twice yearly in 10.5% of the dogs. Over 45% of the dogs had not been dewormed in more than a year. None of the studied dogs were under continuous prophylaxis against phlebotomine sand flies. Flea and tick prevention (repellent or insecticidal treatments) was administered following the manufacturer's recommendations in 34% of the owned dogs, while 60.7% were overdue for flea and ticks’ prevention and 1.8% had never received any (Additional file 3: Table S2).

In the feline group, 3/33 (10%) cats presented at least two clinical manifestations suggestive of FeL, including alopecia (100%), cachexia (66.7%), seborrhea (66.7%), hyperkeratosis, (33.3%), crusts (66.7%) and conjunctivitis (33.3%), and were classified as “FeL-suspect” (Additional file 6: Table S5).

### PCR and DNA sequencing

Amplification of *Leishmania* kDNA identified infection among 19.4% of the dogs (30/155) (Additional file 7: Table S6). Highest recorded infection determined by kDNA-PCR positivity was found in sheltered dogs (24.5%; 24/98 vs. 10.5%; 6/57 domestic dogs). Overall, kDNA positivity detected in dogs from Rabat and Fez was similar (18.8%; 15/80 vs. 20.0%; 15/75) (Additional file 7: Table S6). Four feline genomic DNA samples tested positive for *Leishmania* kDNA-PCR (4/32; 12.5%).

The majority (*n* = 27/30) of the kDNA-positive canine samples tested negative for ITS-1 and SSUrNA. Only three samples were positive for ITS1-PCR and/or SSUrRNA-LnPCR, and sequencing was possible. MOR.DOG.14 was positive for both ITS-1 (GeneBank: PP905395) and SSUrRNA-LnPCR (GeneBank: PP905236). MOR.DOG.105 was positive for SSUrRNA-LnPCR (GeneBank: PP905254) and MOR.DOG.84 was positive for ITS1-PCR (GeneBank: PP905552). To search for similarities, multiple sequence alignments of nt datasets were performed. The highest degree of similarity (100% query cover and 100% identity) was found only for *L. infantum* for both ITS-1 sequences. Concerning SSUrRNA, the highest degree of similarity (99% query cover and 99.72% identity) was found for the *Leishmania donovani* complex.

All four kDNA-positive feline samples tested negative by *Leishmania* ITS1-PCR and SSUrRNA-LnPCR. Still, an attempt to identify the infecting *Leishmania* spp. was carried out by sequencing the purified amplicons obtained in positive kDNA samples (Additional file 8: Table S7). Three out of the four kDNA-PCR positive samples had sufficient DNA concentration and quality for sequencing. The search for similarities was performed by multiple sequence alignments with nt datasets and the results were confirmatory of infection with *Leishmania* spp.

With the goal of understanding bias-related sample collection, statistical analysis was performed among five variables, including sex, breed, housing, city and presence of clinical manifestations suggestive of leishmaniosis (Additional file 7: Table S6). The animal’s age was excluded from the analysis because most of the sampled animals (92.3%) were adult dogs. No significance was attributed to differences obtained by kDNA-PCR positivity and the dog's sex, breed, city and housing conditions. Yet, as expected, statistically significant differences (*p* = 0.028) were detected between kDNA-PCR positivity and the presence of clinical manifestations suggestive of CanL. Among CanL clinically suspect dogs, 30.6% tested kDNA-PCR positive. Subclinical infection was diagnosed among 14.2% of the non-CanL suspect dogs (Additional file 7: Table S6). From this analysis, we excluded possible sample bias and conclude that CanL burden is as serious in Rabat as in Fez.

Feline infection with *Leishmania* spp. (determined by kDNA-PCR positivity) was also subject to statistical analysis by Fisher’s exact test (Table 1). Four variables were included: sex, age group, habitat (being indoor or having outdoor access, herein representing stray cats and owned cats with outdoor access) and the presence of clinical manifestations suggestive of leishmaniosis. Breed was excluded since all cats were domestic short hair. All four kDNA-PCR-positive cats were adult females living outdoors, three of which presented no clinical manifestations suggestive of FeL. No statistically significant differences were observed between kDNA positivity and any of the defined variables (Table 1).

**Table 1:** Prevalence of *Leishmania* spp. in 32 cats from Rabat, Morocco, as determined by polymerase chain reaction (PCR) to kinetoplast DNA, according to categories of independent variables sex, age group, habitat and presence of clinical manifestations compatible with feline leishmaniosis (FeL)

Variable/ category	No. (%) of cats tested	Percentage (*n*) of PCR positive	95% CI (%)^a^
Sex	30 (93.8)	*p* = 0.548^b^	
Female	23 (71.9)	17.4 (4)	5.0–38.8
Male	7 (21.9)	0.0 (0)	0.0–41.0
Age group	30 (93.8)	*p* = 1.0^b^	
< 1 year	2 (6.3)	0.0 (0)	0.0–84.2
1–9 years	28 (87.5)	14.3 (4)	4.0–32.7
Habitat	32 (100)	*p* = 1.0^b^	
Indoors	5 (15.6)	0.0 (0)	0.0–52.2
Outdoors	27 (84.4)	14.8 (4)	4.2–33.7
FeL clinical manifestations^c^	32 (100)	*p* = 0.340^b^	
Absent	29 (90.6)	10.3 (3)	2.2–27.4
Present	3 (9.4)	33.3 (1)	0.8–90.6
Total	32 (100)	12.5 (4)	3.5–29.0

^a^95% confidence interval

^b^Fisher’s exact test

^c^Two or more clinical manifestations compatible with FeL comprising generalized alopecia, seborrhea, crusts, conjunctivitis, cachexia and hyperkeratosis

### Seropositivity to three different *Leishmania*-specific antigens and its relation to demographic characteristics, clinical manifestations of leishmaniosis and kDNA-PCR positivity

Canine seropositivity assessed by ELISA ranged from 17 to 52%, according to the studied antigens (35.2%, 44/125 SPLA; 16.8%, 21/125 rKDDR; 52%, 65/125 LicTXNPx) (Table 2). On the other hand, all feline sera (25/33) tested for anti-*Leishmania* total IgG by means of IFAT were negative at 1:80 dilution.

**Table 2:** Prevalence of *Leishmania* spp. in 125 dogs from Rabat and Fez, Morocco, as determined by enzyme immunosorbent assay (ELISA) antigens soluble promastigote *Leishmania* antigens (SPLA), *Leishmania infantum* recombinant kinesin degenerated derived repeat (rKDDR) and recombinant cytosolic peroxiredoxin protein (LicTXNPx), according to categories of independent variables sex, age group, habitat, presence of clinical manifestations compatible with canine leishmaniosis (CanL) and positivity to kinetoplast DNA polymerase chain reaction (kDNA-PCR)

Variable/ category	No. (%) of dogs tested	Percentage (*n*) of SPLA positive	Percentage (*n*) of rKDDR positive	Percentage (*n*) of LicTXNPx positive
Sex	123 (98.4)	*p* = 0.885	*p* = 0.728	*p* = 0.503
Female	69 (56.1)	36.2 (25)	18.8 (13)	47.8 (33)
Male	54 (43.9)	33.3 (18)	14.8 (8)	55.6 (30)
Breed	123 (98.4)	*p* = 0.665	*p* = 0.601	*p* = 1.0
Mongrel	112 (91.1)	33.9 (38)	16.1 (18)	50.9 (57)
Defined/crossbreed	11 (8.9)	45.5 (5)	27.3 (3)	54.5 (6)
Housing	125 (100)	*p* = 0.448	*p* = 0.205	*p* = 0.061
Shelter	90 (72.0)	37.8 (34)	20.0 (18)	57.8 (52)
Domestic	35 (28.0)	28.6 (10)	8.6 (3)	37.1 (13)
City	125 (100)	*p* = 0.021*	*p* < 0.001*	*p* = 0.025*
Rabat	78 (62.4)	26.9 (21)	6.4 (5)	43.6 (34)
Fez	47 (37.6)	48.9 (23)	34.0 (16)	66.0 (31)
Clinical manifestations of CanL	125 (100)	*p* = 0.001*	*p* < 0.001*	*p* = 0.057
Absent	80 (64.0)	23.8 (19)	3.8 (3)	45.0 (36)
Present^a^	45 (36.0)	55.6 (25)	40.0 (18)	64.4 (29)
kDNA-PCR	125 (100)	*p* = 0.019*	*p* = 0.001*	*p* = 0.061
Positive	29 (23.2)	55.2 (16)	37.9 (11)	69.9 (20)
Negative	96 (76.8)	29.2 (28)	10.4 (10)	46.9 (45)
Total	100.0 (125)	35.2 (44)	16.8 (21)	52.0 (65)

^a^Two or more clinical manifestations compatible with CanL, comprising: kidney disease, anaemia, fever, cachexia, ophthalmic disorders, onychogryphosis, skin disorders, lymphadenopathy

^*^Statistically significant difference (*p* < 0.05)

**Fig. 1:**
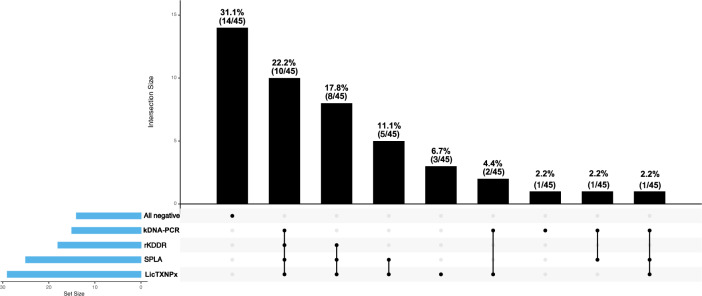
Upset plot depicting intersection of positive results for polymerase chain reaction (PCR), *Leishmania* kinesin degenerated-derived repeats (rKDDR), soluble promastigote *Leishmania* crude proteins (SPLA) and *Leishmania infantum* cytosolic tryparedoxin peroxidase (LicTXNPx) in the group of dogs with canine leishmaniosis clinical manifestations (*n =* 45). The vertical columns in the graph represent the absolute number and percentage of positive events of each intersection, associated with the four parameters evaluated. The connecting line below each column represents the intersection of the tested parameters. The absence of an intersecting line means positivity to one event (quantified in the upper black column)

Considering CanL suspect dogs (*n =* 45), the diagnosis was supported by at least one laboratory test in 21 dogs (68.9%) (Fig. 1). Only one CanL suspect dog tested positive on kDNA-PCR and negative for any *Leishmania*-specific ELISA antigen. Paired positivity to kDNA-PCR and all *Leishmania*-specific antigens confirmed the diagnosis in 22.2% (10/45) of CanL suspect cases. Positivity to any *Leishmania*-specific ELISA antigens in the presence of a negative kDNA-PCR result confirmed 35.5% (16/45) of CanL suspicions. This was detected by single-antigen positivity (6.7%, 3/45), double-antigen positivity (11.1%, 5/45) or triple-antigen positive combinations (17.8%, 8/45) (Fig. 1).

**Fig. 2:**
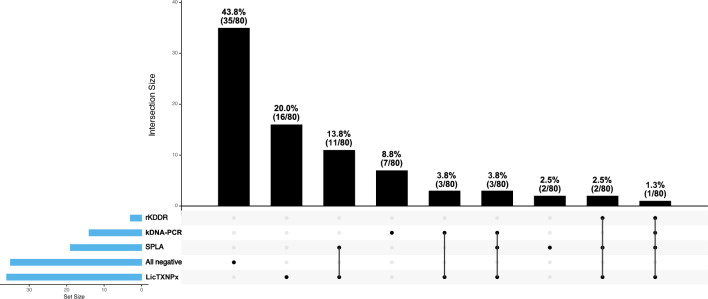
Upset plot depicting intersection of positive results for polymerase chain reaction (PCR), *Leishmania* kinesin degenerated-derived repeats (rKDDR), soluble promastigote *Leishmania* crude proteins (SPLA) and *Leishmania infantum* cytosolic tryparedoxin peroxidase (LicTXNPx) in the group of dogs without canine leishmaniosis clinical manifestations (*n =* 80). The vertical columns in the graph represent the absolute number and percentage of positive events of each intersection associated with the four parameters evaluated. The connecting line below each column represents the intersection of the tested parameters. The absence of an intersecting line means positivity to one event (quantified in the upper black column)

Among dogs without clinical manifestations of CanL (*n =* 80), subclinical infection was supported by at least one laboratory test in 45 animals (56.3%) (Fig. 2). Single kDNA-PCR positivity confirmed infection in seven cases of subclinical CanL (8.8%), while the combination of kDNA-PCR and at least one ELISA antigen was positive for another seven non-suspect CanL (8.8%) (Fig. 2). Thirty-two dogs (40.0%) tested negative on kDNA-PCR but positive for at least one *Leishmania*-specific ELISA antigen; among these, 20% were seropositive to LicTXNPx alone (16/80), and 13.8% (11/80) combined seropositivity to SPLA and LicTXNPx and 3.8% (3/80) seropositivity to all three *Leishmania*-specific antigens (Fig. 2).

To evaluate the differences between techniques, kDNA-PCR positivity and seropositivity against any of the *Leishmania*-specific antigens was analysed by the McNemar test (paired samples). Statistically significant differences (*p* < 0.05) were detected between kDNA-PCR and the different ELISA techniques but also between seropositivity to the different ELISA *Leishmania*-specific antigens (Table 2). The best agreement between kDNA-PCR and ELISA positivity was seen for rKDDR (*κ* = 0.304), following kDNA-PCR and SPLA (*κ* = 0.220) and kDNA-PCR and LicTXNPx (*κ* = 0.154). Among the three different *Leishmania*-specific antigens, moderate agreement was found for SPLA and LicTXNPx (*κ* = 0.575) followed by SPLA and rKDDR (*κ* = 0.543) (Table 3).

**Table 3:** Comparison of polymerase chain reaction (PCR), soluble promastigote *Leishmania* antigens (SPLA), *Leishmania infantum* recombinant kinesin degenerated derived repeat (rKDDR) and recombinant cytosolic peroxiredoxin protein (LicTXNPx) results by Chi-squared (*χ*^2^) or Fisher’s exact (FET), McNemar and Cohen’s kappa coefficient (κ) tests in 125 dogs from Rabat and Fez, Morocco

PCR	PCR			
SPLA	McNemar, *P* = 0.028* κ = 0.220, *P* = 0.010*	SPLA		
rKDDR	McNemar, *P* = 0.185 κ = 0.304, *P* < 0.001*	McNemar, *P* < 0.001* κ = 0.543, *P* < 0.001*	rKDDR	
LicTXNPx	McNemar, *P* = 0.001* κ = 0.154, *P* = 0.037*	McNemar, *P* < 0.001* κ = 0.575, *P* < 0.001*	McNemar, *P* < 0.001* κ = 0.316, *P* < 0.001*	LicTXNPx

^*^Statistically significant difference

Cohen’s kappa coefficient (κ) interpretation: < 0 no agreement; 0.0–0.20 slight agreement, 0.21–0.40 fair agreement, 0.41–0.60 moderate agreement, 0.61–0.80 substantial agreement, 0.81–1.0 almost perfect agreement

Statistically significant differences were identified between seropositivity to any *Leishmania*-specific antigen and the dog's city of origin (Rabat vs. Fez) and between seropositivity to SPLA or rKDDR and the presence of clinical manifestations of CanL. The remaining studied variables, namely sex, breed and housing conditions, were not associated with increased seropositivity to any of the studied *Leishmania*-specific antigens (Table 2).

### Veterinary survey results

#### Participants' demographic and professional characterization

In total, 50/220 contacted veterinarians have fulfilled the questionnaire (23% response). All represented veterinarians graduated from the same university (IAV Hassan II, Rabat, Morocco). Female veterinarians accounted for 60% (30/50) of the responding veterinarians. The median age of the group was 26 (24–65) years. Most of the respondents (80%, 44/50) were practicing veterinary medicine for up to 5 years and worked in urban settings (44 urban practice vs. 8 rural practice). Exclusive small animal practice comprised 68% (34/50) of the clinicians, followed by 26% in mixed (13/50) and 4% in large animal practice (2/50). Responding veterinarians were based in 28 different cities distributed through 11 out of 12 regions of Morocco (Additional file 9: Table S8). The majority was based in Rabat-Salé-Kénitra (*n* = 18, 36.7%) and Casablanca-Settat (*n* = 16; 32.7%) states, followed by Fès-Meknès (*n* = 4; 8.2%), Soouss-Massa (*n* = 3; 2.0%), Marrakesh-Safi (*n* = 3; 6.1%), Tanger-Tetouan-Al Hoceima (*n* = 2; 4.1%), Béni Mellal-Khénifra (*n* = 1; 2%), Laâyoune-Sakia El Hamra (*n* = 1; 2.0%), Oriental (*n* = 1; 2.0) and Dakhla-Oued Ed-Dahab (*n* = 1; 2.0%) state provinces (Additional file 9: Table S8).

### Results on veterinarians’ general knowledge of leishmaniosis epidemiology in Morocco

Answers to individual questions regarding Moroccan veterinarians’ general knowledge of leishmaniosis epidemiology in the country are summarized in Additional file 10: Table S9. Most of the questioned veterinarians (74%) recognized leishmaniosis as a disease of zoonotic potential, with vector-borne origin (identified by 98% of the respondents) and having phlebotomine sandflies as the most important natural vectors in Morocco (identified by 90% of the respondents). Likewise, 76% correctly identified *L. infantum* as the causative agent of canine and feline leishmaniosis, and 54% recognized dogs as the primary host for this parasite. Regarding other *Leishmania* spp. of public health importance and endemic in Morocco, 24% of the veterinarians were aware that desert rats (genus *Meriones*) are primary host of *L. major*, and 20% positively associated humans as reservoirs of *L. tropica*. When questioned about the most important reservoir host for *L. major* and *L. tropica*, 32 and 44% of the respondents replied “don't know”, respectively.

### Results on veterinary knowledge and practices regarding leishmaniosis diagnosis, treatment and prevention

Answers to individual questions inquiring about Moroccan veterinarian’s clinical experience with animal leishmaniosis (including previous diagnosis, treatment and current prophylactic recommendations) are summarized in Table 4 and Additional file 11: Table S10. Thirty-seven (74%) of the questioned veterinarians mentioned having diagnosed animal leishmaniosis in the past, while 12 clinicians (24%) had never done so. Canine leishmaniosis was diagnosed by 36 (71%) of the veterinarians, and 1 veterinarian diagnosed leishmaniosis in a donkey. Among those who had diagnosed leishmaniosis in the past, 76% (28/37) correctly prescribed at least one anti-leishmanial drug (either allopurinol, meglumine antimoniate or miltefosine) (Table 4 and Additional file 11: Table S10). However, the ability to diagnose and treat leishmaniosis was not associated with clinical experience, as no statistically significant differences were observed between previous diagnosis of animal leishmaniosis, years of clinical practice (*p* = 0.086) or correct prescription of at least one specific treatment (Table 4).

**Table 4:** Knowledge and clinical decisions of Moroccan veterinarians (*n =* 50) regarding animal leishmaniosis according to type of practice, experience, practice location and knowledge of guidelines for the management of animal leishmaniosis

Variable/ category	Number (*n*) of	Percentage (%) of positive responses				
	respondents	Previous diagnosis of leishmaniosis	Identification of clinical manifestations (3 or more)^b^	Specific treatment applied^c^	Recommendation of CanL prevention	Knowledge of guidelines^d^
Type of practice	50 (100)	*p* = 1.0^a^	*p* = 1.0^a^	*p* = 0.658^a^	*p* = 1.0^a^	*p* = 1.0^a^
Small animal and mixed	37 (74.0)	73.0 (27)	70.3 (26)	80.8 (21)	37.8 (14)	13.5 (5)
Large animal	13 (26.0)	79.6 (10)	69.2 (9)	70.0 (7)	38.5 (5)	15.4 (2)
Years in practice		*p* = 0.086^a^	*p* = 0.043^a^	*p* = 0.648^a^	*p* = 0.273^a^	*p* = 1.0^a^
Up to 5	41 (82.0)	68.3 (28)	63.4 (26)	74.1 (20)	34.1 (14)	14.6 (6)
More than 5	9 (18.0)	100 (9)	100 (9)	88.9 (8)	55.6 (5)	11.1 (1)
Location		*p* = 0.181^a^	*p* = 0.043^a^	*p* = 1.0^a^	*p* = 0.018^a^	*p* = 0.580^a^
Urban	42 (84.0)	78.6 (33)	76.2 (32)	78.1 (25)	45.4 (19)	16.7 (7)
Rural	8 (16.0)	50.0 (4)	37.5 (3)	75.0 (3)	0.0 (0)	0.0 (0)
Knowledge of guidelines^d^	50 (100)	*p* = 0.660^a^	*p* = 0.659^a^	*p* = 0.302^a^	*p* = 0.404^a^	–
Yes	7 (14.0)	85.7 (6)	85.7 (6)	100.0 (6)	57.1 (4)	–
No	33 (66.0)	72.1 (31)	67.4 (29)	73.3 (22)	34.9 (15)	–
Total	50 (100)	74.0 (37)	70.0 (35)	77.8 (28)^e^	38.0 (19)	14.0 (7)

^a^Fisher’s exact test

^b^Comprising lymphadenopathy, skin disorders, onychogryphosis, ophthalmic disorders, cachexia, fever, anemia and kidney disease

^c^Antileishmanial treatments applied include sole or combination of leishmanicidal (miltefosine, meglumine antimoniate) and leishmaniostatic (allopurinol) drugs

^d^LeishVet, ESCCAP (European Scientific Counsel Companion Animal Parasites) or CLWG (Canine Leishmaniasis Working Group) guidelines; ^e^computed for 36 responses only

Fourteen percent (7/50) of the respondents reported being familiar with international guidelines for animal leishmaniosis, such as the LeishVet, ESCCAP (European Scientific Counsel Companion Animal Parasites) or CLWG (Canine Leishmaniasis Working Group) recommendations (Table 4 and Additional file 11: Table S10). No statistically significant differences (*p* > 0.05) were found between knowledge of guidelines for clinical leishmaniosis and having diagnosed leishmaniosis in the past, describing its clinical manifestations (list three or more clinical signs) and treat the disease (Table 4).

When requested to describe the clinical manifestations of leishmaniosis identified in previously diagnosed cases, 70% (35/50) of the clinicians correctly identified and described three or more clinical signs, 6% (3/50) described at least two clinical signs and only 4% (2/50) described one (Additional file 11: Table S10). The ability to describe three or more CanL clinical manifestations presented statistically significant differences between those practicing for < or > 5 years (*p* = 0.043) and working in urban or rural settings (Table 4).

Regarding prophylactic measures to prevent canine infection by *Leishmania* parasites, 36% (18/50) of the veterinarians reported their recommendation, even if only to animals living outdoors (12/36%; 6/18) (Table 4). Previous knowledge of guidelines for leishmaniosis management had no positive statistical association with prophylactic recommendations for this disease (Table 4). Veterinarians working in urban areas are keener to recommend CanL prophylaxis compared to those working in rural environments (*p* = 0.018).

## Discussion

The first report of CanL in Morocco dates from 1932, and studies on prevalence have been performed since then but irregularly distributed over time and space [11]. In the present study, we investigated, for the first time, canine and feline infection with *Leishmania* spp. in the urban centres of Rabat and Fez. Simultaneously, a questionnaire was distributed among Moroccan veterinarians to assess their knowledge and practices towards leishmaniosis epidemiology in the country as well as clinical management of the disease in animals.

This study revealed that the global molecular prevalence of *Leishmania* in dogs from Rabat and Fez, considering positivity to the kDNA-PCR, was 19.4%. No significant differences in CanL prevalence were observed in both cities, with 18.8% prevalence in Rabat and 20% in Fez. These results are consistent with previous reports estimating a pool CanL prevalence of 29% in the region of Rabat-Sale-Kénitra and 20% in the Fez-Meknes region [11]. The only characteristic that significantly impacted the detection of kDNA was the presence of clinical signs suggestive of CanL. In fact, molecular testing confirmed CanL diagnosis in 30.6% of suspect presentations, while 14.2% of the non-CanL suspect dogs were found subclinically infected. This is in line with other studies reporting increased capacity of PCR to detected *Leishmania* in the presence of clinical signs [23]. Still, detection of DNA in animals without clinical signs is not uncommon [23, 27]. The sequencing of ITS-1-positive products was possible for two dogs, confirming *L. infantum* infection. This observation is expected considering the epidemiological context [5, 11].

In cats, kDNA-PCR detected four positive cases, with one being clinically suspect. This is the first report of possible feline infection with *Leishmania* spp. in Morocco. The prevalence of feline infection as detected by PCR (12.5%; Additional file 9: Table S8) was lower than that of canine infection (19.4%, Additional file 7: Table S6) within the urban setting of Rabat. Likewise, lower prevalence of FeL compared to CanL in regions of high endemicity for leishmaniosis has been reported in other Mediterranean countries such as Greece and Portugal [38–40]. Importantly, the kDNA sequencing supported feline infection with *Leishmania* spp. Although the best identification obtained from sequence alignment matched *L. infantum* kDNA sequences, again expected for the geographical area, the heterogenic nature of minicircle networks hampers the use of this peculiar genomic region for a particular species and strain typing.

Considering individual antigens, the highest percentage of seropositivity was associated with LicTXNPx (52.0%) followed by SPLA (35.2%) and rKDDR (16.8%). Double seropositivity to rKDDR and SPLA was evident in 18.8% of the animals and 16.8% of the samples positive to all ELISA antigens (Figs. 1 and 2). These numbers, using combined seropositivity to rKDDR and SPLA as antigens, fit the available epidemiological data from Morocco. Moreover, seropositivity to SPLA and rKDDR was significantly more overrepresented in clinically suspect animals, supporting the capacity of the presented cut-offs for these two antigens to detect disease and provide relevant epidemiological data in this geographical context. High seropositivity to LicTXNPx contrasts with available epidemiological information on the region and can be justified by the facts that the cut-offs were calculated using cohorts from other geographical regions and that maybe some environmental organism may be generating cross-reactivity to this antigen. The fact that LicTXNPx was not significantly overrepresented in clinically suspect dogs, unlike rKDDR or SPLA, at first glance could be considered indicative of cross-reactivity with other pathogens. Still, LicTXNPx was previously associated with early seroconversion in experimentally infected dogs and was able to detect subclinical infections [34].

In the present study, no statistically significant differences were identified between kDNA-PCR positivity (Additional file 7: Table S6) or seropositivity to any *Leishmania*-specific antigen (Table 1) when comparing owned and sheltered dogs from both cities, although global percentual seropositivity to any antigen was found higher in sheltered dogs compared to domestic. Importantly, none of the 156 dogs were undergoing continuous and effective prophylaxis against CanL, a circumstance which may have increased the risk to vector exposure and *Leishmania* infection. Despite a lack of sufficient studies that simultaneously compare CanL prevalence between owned and sheltered/stray dogs from the same region, the hypothesis that stray and sheltered animals are more prone to *Leishmania* infection compared to companion dogs has been supported [41] and contradicted [42]. Such differences can be explained by the level of veterinary care and CanL prophylaxis measures provided to each group of animals [42].

Concerning cats, although four animals were PCR positive for *Leishmania* kDNA, lack of agreement between molecular and serological detection of FeL has been previously reported [43, 44]. Overall, the molecular data generated suggest an important presence of *Leishmania* in urban settings. Under the current experimental settings, the amplification of ITS-1 and SSUrRNA products out of kDNA-positive feline samples was not productive. This can be justified by the fact that kDNA amplification offers higher sensitivity, since > 10,000 copies of kDNA can be present per parasite, while ITS1 fragments present dozens to over a few hundred copies [45]. Before concluding about cats' participation in the epidemiology of the disease in Morocco, a more extensive approach to feline *Leishmania* infection is required.

For the first time, the present study provides data on the veterinary practitioners’ knowledge of the epidemiology and clinical management of leishmaniosis in Morocco. Two hundred twenty Moroccan veterinarians, enrolled in a previous survey, were electronically contacted with a request to participate in a questionnaire involving questions related to their knowledge, perceptions and practices regarding zoonotic diseases. Fifty respondents completed the questionnaires. The veterinary survey showed an optimistic scenario regarding veterinary knowledge of animal leishmaniosis epidemiology in Morocco. This is reflected in a high percentage (74%) of the questioned veterinarians who had diagnosed leishmaniosis in the past and prescribed at least one dedicated treatment (76% of those diagnosed leishmaniosis). Compared to Portuguese veterinarians [46], Moroccans are less familiar with international guidelines for CanL and FeL management (with 14% of Moroccan respondents being familiar with such recommendations) (Table 4 and Additional file 11: Table S10). Nevertheless, similarly to a group of surveyed Spanish and French peers, the level of knowledge on international guidelines for CanL and FeL does not impair recognition of clinical signs of the disease or its diagnosing and prescription of a treatment in line with such guidelines [47]. Nonetheless, increasing clinician’s awareness of *Leishmania* spp. infection among other domestic species, namely the cat, would profit the practitioners. An absence of recommendations for an effective prophylaxis against phlebotomine sand fly vectors was found among all respondent veterinarians. Besides, 64% of the questioned clinicians do not recommend CanL prevention and only 12% do so if the animal lives outdoors (Table 4 and Additional file 11: Table S10). Thus, it is important to reinforce this measure among animal health providers to tackle *Leishmania* spp. transmission in Morocco.

## Conclusions

The results of the present study support previous assumptions about the presence of *Leishmania* infections among primary reservoir animals living within urban boundaries of Moroccan cities, with kDNA-PCR pointing to 19.4% prevalence of infection among dogs from the cities of Rabat and Fez and 12.5% in cats from Rabat. Besides, *L. infantum* was confirmed to be the infecting species in two dogs. For the first time, Moroccan veterinarians were surveyed on their knowledge on leishmaniosis epidemiology and clinical decisions regarding its management. From the analyses of the questioned veterinarian cohort, we recognized that Moroccan veterinarians are able to identify and manage CanL. Finally, considering both the epidemiological and veterinary survey findings, it still seems necessary to reinforce recommendations and strategies for effective prophylactic approaches to tackle canine and feline *Leishmania* infection and disease in urban settings of Morocco. Overall, this report highlights the need for a collaborative effort between epidemiological research and veterinary practice to mitigate the impact of leishmaniosis on both animal and human populations in the region.

### Supplementary Information

**Additional file 1: Table S1.** Polymerase chain reaction (PCR) amplification conditions for *Leishmania* spp. kinetoplast DNA (kDNA) and internal transcribed spacer 1 (ITS-1) primers

**Additional file 2: Figure S1.** Veterinary questionnaire.

**Additional file 3: Table S2.** Characterization of the canine study group (*n =* 156): description of number of sampled dogs and their demographic, clinical and prophylactic history.

**Additional file 4: Table S3.** Characterization of the feline study group (*n =* 33): description of number of sampled cats and their demographic, clinical and prophylactic history.

**Additional file 5: Table S4.** Clinical manifestations in 48 dogs suspected of canine leishmaniosis (CanL).

**Additional file 6: Table S5.** Clinical manifestations in three cats suspected of feline leishmaniosis (FeL).

**Additional file 7: Table S6.** Prevalence of *Leishmania* spp. in 155 dogs from Rabat and Fez, Morocco, as determined by polymerase chain reaction (PCR), according to categories of independent variables sex, breed, housing, city and presence of clinical manifestations compatible with canine leishmaniosis (CanL).

**Additional file 8: Table S7.** Partial kinetoplast DNA (kDNA) sequences identified in the three kDNA-positive cats from the study.

**Additional file 9: Table S8.** Demographic characteristics of Moroccan veterinarians participating in the questionnaire.

**Additional file 10: Table S9.** Moroccan veterinarians’ responses to questions related to the epidemiology of leishmaniosis in Morocco (*n =* 50).

**Additional file 11: Table S10.** Moroccan veterinarians’ responses to questions related to clinical management of leishmaniosis (*n =* 50).

## Data Availability

All data and materials are available at the Host-Parasite Interaction Group (I3S). The generated ITS1 sequences of *Leishmania infantum* have been deposited in the GenBank database under accession nos. PP905552 and PP905395. The generated SSUrNA sequences of *L. infantum* have been deposited in the GenBank database under accession nos. PP905254 and PP905236.

## Abbreviations

CanL: Canine leishmaniosis
CL: Cutaneous leishmaniosis
ELISA: Enzyme-linked immunosorbent assay
FeL: Feline leishmaniosis
FET: Fisher's exact test
IAV: Institut Agronomique et Vétérinaire
IgG: Immunoglobulin G
IFAT: Indirect immunofluorescence antibody test
ITS-1: Internal transcribed spacer
kDNA: Kinetoplast DNA
LicTXNPx: *Leishmania** infantum* cytosolic tryparedoxin peroxidase
LnPCR: *Leishmania*-specific nested PCR
nt: Nucleotide
NTD: Neglected tropical diseases
PCR: Polymerase chain reaction
rKDDR: *Leishmania* kinesin degenerated derived repeats
SPLA: Soluble promastigote *Leishmania* crude proteins
SSUrRNA: Small subunit ribosomal RNA
TNR: Trap-neuter-release
VL: Visceral leishmaniosis

## References

[1] Steverding D. The history of leishmaniasis. Parasit Vectors. 2017;10:82. 28202044 10.1186/s13071-017-2028-5 PMC5312593

[2] Álvarez-Hernández D-A, Rivero-Zambrano L, Martínez-Juárez L-A, García-Rodríguez-Arana R. Overcoming the global burden of neglected tropical diseases. Ther Adv Infect Dis. 2020;7:2049936120966449. 33178435 10.1177/2049936120966449 PMC7592315

[3] Tabbabi A. Review of leishmaniasis in the Middle East and North Africa. Afr Health Sci. 2019;19:1329–37. 31148958 10.4314/ahs.v19i1.4 PMC6531937

[4] Aoun K, Bouratbine A. Cutaneous leishmaniasis in North Africa: a review. Parasite. 2014;21:14. 24626301 10.1051/parasite/2014014 PMC3952656

[5] Hakkour M, El Alem MM, Hmamouch A, Rhalem A, Delouane B, Habbari K, et al. Leishmaniasis in northern Morocco: predominance of *Leishmania infantum* compared to *Leishmania tropica*. Biomed Res Int. 2019;2019:5327287. 31485441 10.1155/2019/5327287 PMC6702844

[6] El-Mouhdi K, Chahlaoui A, El Ouali LA, Bouzid J, el Omari H, Mohamed F. Situation épidémiologique des leishmanioses au niveau de la ville d’El Hajeb (centre du Maroc) durant la période de 2013 à 2017. Eur Sci J. 2019;15:155–68.

[7] Hakkour M, Hmamouch A, El Alem MM, Rhalem A, Amarir F, Touzani M, et al. New epidemiological aspects of visceral and cutaneous leishmaniasis in Taza, Morocco. Parasit Vectors. 2016;9:612. 27899126 10.1186/s13071-016-1910-x PMC5129210

[8] El-Mouhdi K, Fekhaoui M, Elhamdaoui F, Guessioui H, Chahlaoui A. Knowledge and experiences of health professionals in the peripheral management of leishmaniasis in Morocco (ELHajeb). J Parasitol Res. 2020;2020:8819704. 33014439 10.1155/2020/8819704 PMC7512069

[9] Burza S, Croft SL, Boelaert M. Leishmaniasis. Lancet. 2018;392:951–70. 30126638 10.1016/S0140-6736(18)31204-2

[10] Bennis I, Thys S, Filali H, De Brouwere V, Sahibi H, Boelaert M. Psychosocial impact of scars due to cutaneous leishmaniasis on high school students in Errachidia province, Morocco. Infect Dis Poverty. 2017;6:46. 28385151 10.1186/s40249-017-0267-5 PMC5383955

[11] El-Mouhdi K, Boussaa S, Chahlaoui A, Fekhaoui M. Prevalence and risk factors of canine leishmaniasis in Morocco: a systematic review and meta-analysis. J Parasit Dis. 2022;46:967–87. 36457764 10.1007/s12639-022-01521-2 PMC9606190

[12] Asmae H, Fatima A, Hajiba F, Mbarek K, Khadija B, Mohamed R, et al. Coexistence of *Leishmania tropica* and *Leishmania infantum* in Sefrou province, Morocco. Acta Trop. 2014;130:94–9. 24161534 10.1016/j.actatropica.2013.10.012

[13] Zarrouk A, Kahime K, Boussaa S, Belqat B. Ecological and epidemiological status of species of the *Phlebotomus perniciosus* complex (Diptera: Psychodidae, Phlebotominae) in Morocco. Parasitol Res. 2016;115:1045–51. 26593735 10.1007/s00436-015-4833-0

[14] Boussaa S, Kahime K, Samy AM, Salem AB, Boumezzough A. Species composition of sand flies and bionomics of *Phlebotomus**papatasi* and *P*. *sergenti* (Diptera: Psychodidae) in cutaneous leishmaniasis endemic foci, Morocco. Parasit Vectors. 2016;9:60. 26830689 10.1186/s13071-016-1343-6 PMC4736259

[15] Quinell RJ, Courtenay O. Transmission, reservoir hosts and control of zoonotic visceral leishmaniasis. Parasitology. 2009;136:1915–34. 19835643 10.1017/S0031182009991156

[16] Kahime K, Boussaa S, Nhammi H, Boumezzough A. Urbanization of human visceral leishmaniasis in Morocco. Parasite Epidemiol Control. 2017;2:1–6. 29774290 10.1016/j.parepi.2017.07.001 PMC5952660

[17] Pennisi MG, Persichetti MF. Feline leishmaniosis: is the cat a small dog? Vet Parasitol. 2018;251:131–7. 29426470 10.1016/j.vetpar.2018.01.012 PMC7130840

[18] Pereira A, Maia C. *Leishmania* infection in cats and feline leishmaniosis: an updated review with a proposal of a diagnosis algorithm and prevention guidelines. Curr Res Parasitol Vector Borne Dis. 2021;1:100035. 35284863 10.1016/j.crpvbd.2021.100035 PMC8906079

[19] Vioti G, da Silva MD, Galvis-Ovallos F, Alves ML, da Silva DT, Leonel JAF, et al. Xenodiagnosis in four domestic cats naturally infected by *Leishmania infantum*. Transbound Emerg Dis. 2022;69:2182–90. 34229362 10.1111/tbed.14216

[20] Palatnik-de-Sousa CB, Day MJ. One Health: the global challenge of epidemic and endemic leishmaniasis. Parasit Vectors. 2011;4:197. 21985335 10.1186/1756-3305-4-197 PMC3214158

[21] Müller A, Montoya A, Escacena C, dela Cruz M, Junco A, Iriso A, et al. Leishmania infantum infection serosurveillance in stray dogs inhabiting the Madrid community: 2007–2018. Parasit Vectors. 2022;15:96. 35422058 10.1186/s13071-022-05226-6 PMC9281004

[22] Kaye P, Scott P. Leishmaniasis: complexity at the host-pathogen interface. Nat Rev Microbiol. 2011;9:604–15. 21747391 10.1038/nrmicro2608

[23] Lima CS, Esteves S, Costa I, Brancal H, Lima C, Amorim C, et al. Use of antigen combinations to address complex *Leishmania*-seropositivity patterns in dogs living in canine leishmaniosis endemic regions of Portugal. Microorganisms. 2022;10:2018. 36296294 10.3390/microorganisms10102018 PMC9607924

[24] Sousa S, Lopes AP, Cardoso L, Silvestre R, Schallig H, Reed SG, et al. Seroepidemiological survey of *Leishmania infantum* infection in dogs from northeastern Portugal. Acta Trop. 2011;120:82–7. 21741348 10.1016/j.actatropica.2011.06.003

[25] Santarém N, Sousa S, Amorim CG, de Carvalho NL, de Carvalho HL, Felgueiras Ó, et al. Challenges in the serological evaluation of dogs clinically suspect for canine leishmaniasis. Sci Rep. 2020;10:3099. 32080327 10.1038/s41598-020-60067-6 PMC7033258

[26] Lima C, Mesquita JR, Brancal H, Vahlenkamp T, Teixeira AR, Cardoso L, et al. The use of *Escherichia coli* total antigens as a complementary approach to address seropositivity to *Leishmania* antigens in canine leishmaniosis. Parasitology. 2017;144:1384–93. 28534448 10.1017/S0031182017000713

[27] Correia B, Magalhães A, Rocha L, Cardoso I, Ferreira RRF, Mesa-Sanchez I. Prevalence of subclinical infectious agents in a blood donor population tested on every donation. J Small Anim Pract. 2024;65:176–80. 38185815 10.1111/jsap.13698

[28] Baxarias M, Jornet-Rius O, Donato G, Mateu C, Alcover MM, Pennisi MG, et al. Signalment, immunological and parasitological status and clinicopathological findings of *Leishmania*-seropositive apparently healthy dogs. Animals. 2023;13:1649. 37238079 10.3390/ani13101649 PMC10215614

[29] Santarém N, Silvestre R, Cardoso L, Schallig H, Reed SG, Cordeiro-da-Silva A. Application of an improved enzyme-linked immunosorbent assay method for serological diagnosis of canine leishmaniasis. J Clin Microbiol. 2010;48:1866–74. 20164286 10.1128/JCM.02402-09 PMC2863945

[30] LeishVet. Canine and feline leishmaniosis: a brief for the practicing veterinarian. 2022. https://www.leishvet.org/wp-content/uploads/2023/01/ALIVE-dec22-web-EN.pdf, 2018. Accessed 15 Apr 2024.

[31] Abramo F, Albanese F, Gattuso S, Randone A, Fileccia I, Dedola C, et al. Skin lesions in feline leishmaniosis: a systematic review. Pathogens. 2021;10:472. 33924616 10.3390/pathogens10040472 PMC8070508

[32] Pimenta P, Alves-Pimenta S, Barros J, Barbosa P, Rodrigues A, Pereira MJ, et al. Feline leishmaniosis in Portugal: 3 cases (year 2014). Vet Parasitol Reg Stud Rep. 2015;1–2:65–9. 10.1016/j.vprsr.2016.02.003 31018412

[33] Lachaud L, Marchergui-Hammami S, Chabbert E, Dereure J, Dedet JP, Bastien P. Comparison of six PCR methods using peripheral blood for detection of canine visceral leishmaniasis. J Clin Microbiol. 2002;40:210–5. 11773118 10.1128/JCM.40.1.210-215.2002 PMC120090

[34] Schönian G, Nasereddin A, Dinse N, Schweynoch C, Schallig H, Presber W, et al. PCR diagnosis and characterization of *Leishmania* in local and imported samples. Diagn Microbiol Infect Dis. 2003;47:349–58. 12967749 10.1016/S0732-8893(03)00093-2

[35] Cruz I, Cañavate C, Rubio JM, Morales MA, Chicharro C, Laguna F, et al. A nested polymerase chain reaction (Ln-PCR) for diagnosing and monitoring *Leishmania infantum* infection in patients co-infected with human immunodeficiency virus. Trans R Soc Trop Med Hyg. 2002;96:S185–9. 12055836 10.1016/S0035-9203(02)90074-X

[36] Iatta R, Trerotoli P, Lucchese L, Natale A, Buonavoglia C, Nachum-Biala Y, et al. Validation of a new immunofluorescence antibody test for the detection of *Leishmania infantum* infection in cats. Parasitol Res. 2020;119:1381–6. 32107620 10.1007/s00436-020-06627-1

[37] Lima CM, Santarém N, Neves NC, Sarmento P, Carrapato C, de Sousa R, et al. Serological and molecular survey of *Leishmania infantum* in a population of Iberian lynxes (*Lynx pardinus*). Microorganisms. 2022;10:2447. 36557700 10.3390/microorganisms10122447 PMC9788222

[38] Diakou A, Papadopoulos E, Lazarides K. Specific anti-*Leishmania* spp. antibodies in stray cats in Greece. J Feline Med Surg. 2009;11:728–30. 19254858 10.1016/j.jfms.2008.01.009 PMC11132581

[39] Cardoso L, Lopes AP, Sherry K, Schallig H, Solano-Gallego L. Low seroprevalence of *Leishmania infantum* infection in cats from northern Portugal based on DAT and ELISA. Vet Parasitol. 2010;174:37–42. 20851524 10.1016/j.vetpar.2010.08.022

[40] Maia C, Gomes J, Cristóvão J, Nunes M, Martins A, Rebêlo E, et al. Feline *Leishmania* infection in a canine leishmaniasis endemic region, Portugal. Vet Parasitol. 2010;174:336–40. 20869810 10.1016/j.vetpar.2010.08.030

[41] Cortes S, Afonso MO, Alves-Pires C, Campino L. Stray dogs and leishmaniasis in urban areas, Portugal. Emerg Infect Dis. 2007;13:1431–2. 18252134 10.3201/eid1309.070101 PMC2857284

[42] Afonso P, Coelho AC, Quintas H, Cardoso L. *Leishmania* seroprevalence in dogs: comparing shelter and domestic communities. Animals. 2023;13:2352. 37508129 10.3390/ani13142352 PMC10376450

[43] Spada E, Canzi I, Baggiani L, Perego R, Vitale F, Migliazzo A, et al. Prevalence of *Leishmania infantum* and co-infections in stray cats in northern Italy. Comp Immunol Microbiol Infect Dis. 2016;45:53–8. 27012922 10.1016/j.cimid.2016.03.001 PMC7132376

[44] Maia C, Nunes M, Campino L. Importance of cats in zoonotic leishmaniasis in Portugal. Vector Borne Zoonotic Dis. 2008;8:555–9. 18471058 10.1089/vbz.2007.0247

[45] Bensoussan E, Nasereddin A, Jonas F, Schnur LF, Jaffe CL. Comparison of PCR assays for diagnosis of cutaneous leishmaniasis. J Clin Microbiol. 2006;44:1435–9. 16597873 10.1128/JCM.44.4.1435-1439.2006 PMC1448629

[46] Monteiro M, Prata S, Cardoso L, Pereira da Fonseca I, Leal RO. Diagnosis and clinical management of canine leishmaniosis by general veterinary practitioners: a questionnaire-based survey in Portugal. Parasit Vectors. 2021;14:306. 34099039 10.1186/s13071-021-04799-y PMC8182999

[47] Le Rutte EA, van Straten R, Overgaauw PAM. Awareness and control of canine leishmaniosis: a survey among Spanish and French veterinarians. Vet Parasitol. 2018;253:87–93. 29605010 10.1016/j.vetpar.2018.01.013

