# Supplementary material for: Veterinary perspectives on the urbanization of leishmaniosis in Morocco
Source: Parasit Vectors. 2024 Aug 19;17:348. doi: 10.1186/s13071-024-06411-5 (PMC11334585; doi:10.1186/s13071-024-06411-5)
Supplement: Supplementary file 1 — Additional file 1: Table S1. Polymerase chain reaction (PCR) amplification conditions for Leishmania spp. kinetoplast DNA (kDNA) and internal transcribed spacer 1 (ITS-1) primers [file 13071_2024_6411_MOESM1_ESM.docx]

**Additional file 1: Table S1.** Polymerase chain reaction (PCR) amplification conditions for *Leishmania* spp. kinetoplast DNA (kDNA) and internal transcribed spacer 1 (ITS-1) primers.

| **PCR** | **Primer set** | **Reaction**  **setup** | **Initial denaturation** | **Cycling**  **protocol** | **N. cycles** | **Final elongation** | **Product size (bp)** | **References** |
| --- | --- | --- | --- | --- | --- | --- | --- | --- |
| kDNA | RV1  RV2 | 25 μL reaction volume; 5 μL DNA; 2.5 μL PCR 10× buffer; 0.5 μL dNTP mix (10 mM of each dNTP; dNTP NZYmix, NZYTech, Portugal); 0.75 μL of 50 mM MgCl2; 0.1 μL Taq DNA polymerase (Invitrogen Taq DNA Polymerase Recombinant, 5 U/μL, Carlsbad, CA, USA). 0.8 μM of each primer | 94 ^º^C  3 min | 94◦C for 45sec  Annealing 59 ^º^C  Extension 50sec 72 ^º^C | 40 | 72 ^º^C  10 min | 145 bp | [33] |
| ITS-1 | LiTSR  L5.8S | 25 μL reaction volume; 5 μL DNA; 2.5 μL PCR 10× buffer; 0.5 μL dNTP mix (10 mM of each dNTP; dNTP NZYmix, NZYTech, Portugal); 0.75 μL 50 mM MgCl2; 0.1 μL Taq DNA polymerase (Invitrogen Taq DNA Polymerase Recombinant, 5 U/μL, Carlsbad, CA, USA). 0.8 μM of each primer | 94^0^C  3 min | 94◦C for 45 sec  Annealing 53 ^º^C  Extension 50 sec 72 ^º^C | 35 | 72 ^º^C  10 min | 300  to 350 bp | [34] |
| SSUrRNA  (Ln-PCR) | R221  R332 | 25 μL reaction volume; 5 μL DNA; 2.5 μL PCR 10× buffer; 0.5 μL dNTP mix (10 mM of each dNTP; dNTP NZYmix, NZYTech, Portugal); 0.75 μL 50 mM MgCl2; 0.1 μL Taq DNA polymerase (Invitrogen Taq DNA Polymerase Recombinant, 5 U/μL, Carlsbad, CA, USA). 1.2 μM of each primer | 94 ^º^C  3 min | 94◦C for 3 sec  Annealing 55^0^C 30 sec  Extension 72 ^º^C 80 sec | 35 | 72 ^º^C  10 min | 603 bp | [35] |
|  | R223  R333 | 25 μL reaction volume; 5 μL of the first PCR product diluted 1/25 in nuclease-free water; 2.5 μL PCR 10× buffer; 0.5 μL dNTP mix (10 mM of each dNTP; dNTP NZYmix, NZYTech, Portugal); 0.75 μL 50 mM MgCl2; 0.1 μL Taq DNA polymerase (Invitrogen Taq DNA Polymerase Recombinant, 5 U/μL, Carlsbad, CA, USA). 0.3 μM of each primer | 94 ^º^C  3 min | 94◦C for 30 sec  Annealing 60 ^º^C 30 sec  Extension 72 ^º^C 50 sec | 35 | 72 ^º^C  10 min | 358 bp |  |
