# Supplementary material for: Veterinary perspectives on the urbanization of leishmaniosis in Morocco
Source: Parasit Vectors. 2024 Aug 19;17:348. doi: 10.1186/s13071-024-06411-5 (PMC11334585; doi:10.1186/s13071-024-06411-5)
Supplement: Supplementary file 2 — Additional file 2: Figure S1. Veterinary questionnaire. [file 13071_2024_6411_MOESM2_ESM.pdf]

Additional file 2: Figure S1. Veterinary questionnaire.

Thank you for participating in this questionnaire. This is a **voluntary, confidential** and **anonymous** activity**.** The information gathered below will be used for research purposes focused on animal diseases and the role of veterinary professionals in management. This survey will take approximately 15 min to be completed. Thank you for your time and collaboration.

**Section I – Demographic identification**			
**Please tick (√) or fill in the blank**			
1. Age:			
2. Nationality	☐ Moroccan	☐ Other :	
3. Gender:	☐ male	☐ female	☐ rather not say
4. Background education	☐ Barchelor on Animal Sciences<br>☐ Degree in Veterinary Medicine<br>☐ Masters Degree<br>☐ PhD		
5. Practice location	Urban (city:) ; Rural (location:)		
6. Years in practice	☐ Less than 2yrs<br>☐ Between 2 and 5 yrs<br>☐ Between 5 and 15 yrs<br>☐ Over 15 yrs		
7. Type of practice	☐ Small Animal Clinic<br>☐ Exotic Animals Clinic<br>☐ Large Animals Clinic - Ruminants<br>☐ Large Animals Clinic - Horses<br>☐ Mixed Small Animals and Large Animal Practice<br>☐ Retired<br>☐ Academic<br>☐ Veterinarian teaching and practicing at a university hospital/ referral hospital		
**Section II – Veterinarians awareness of leishmaniosis**			

**Please tick (√) or fill in the blank**		
8. Do you consider leishmaniosis a zoonotic disease?	☐ Yes<br>☐ No	
9. Are you familiar international guidelines for leishmaniosis (e.g ESCCAP, LeishVet or C.L.W.G)?	☐ Yes<br>☐ No	
10. Have you diagnosed leishmaniosis in dogs, cats, horses or donkeys?	☐ Yes, in dogs only<br>☐ Yes, in cats only<br>☐ Yes, in dogs and cats<br>☐ Yes, in horses<br>☐ Yes, in donkeys<br>☐ Yes, in both horses and donkeys<br>☐ No, I have never diagnoses leishmaniosis in any of my patients	
11. If your answer to previous question was **no**, please	☐ There is no animal leishmanisasis in Morroco<br>☐ I am only awaere of human leishmaniosis in Morroco<br>☐ I don't know what leishmaniosis is<br>☐ Morroco is not endemic for leishmaniosis	
12. If the answer to question 1**0** was **yes**, what are the clinical signs you aften associate to animal leishmaniosis?	☐ Fever<br>☐ Anemia<br>☐ Cachexia<br>☐ Lymphadenopathy<br>☐ Onicogryphosis	☐ Renal disease<br>☐ Ophthalmic disorders<br>☐ Dermatological disorders<br>☐ Neurological disorders
13. If the answer to question **20** was **yes**, what where your treatment options?	☐ Alopurinol<br>☐ Domperidone<br>☐ Antimonials<br>☐ Miltefosin<br>☐ Paromomycin<br>☐ Ivermectin	☐ Enrofloxacin<br>☐ Marbofloxacin<br>☐ Doxycicline<br>☐ Imidocarb<br>☐ Amoxicillin<br>☐ Meloxicam

14. Can you identifiy the causative agent of leishmaniosis in dogs and cats?	☐ *Leishmania infantum*<br>☐ *Leishmania tropica*<br>☐ *Leishmania major*<br>☐ I don't know		
15. Can you point the primary reservoir hosts of the following *Leishmania* species?	*Leishmania infantum*<br>☐ Dogs<br>☐ Cats<br>☐ Mice<br>☐ Desert rats<br>☐ Humans<br>☐ I don't know	*Leishmania major*<br>☐ Dogs<br>☐ Cats<br>☐ Mice<br>☐ Desert rats<br>☐ Humans<br>☐ I don't know	*Leishmania tropica*<br>☐ Dogs<br>☐ Cats<br>☐ Mice<br>☐ Desert rats<br>☐ Humans<br>☐ I don't know
16. Can you identify if leishmaniosis is a vector borne disease?	☐ I don't know<br>☐ Not, It is not transmited by a vector<br>☐ Yes, It is transmited by a vector		
17. If your answer to question **16** was **yes**, please identify the vector of transmission.	☐ *Ripicephalus sanguineus* ticks<br>☐ Aedes mosquitos<br>☐ Anopheles mosquitos<br>☐ Phlebotomine sandflies<br>☐ Fleas<br>☐ I know its transmitted by an arthropode vector, but I can not identify which.		

**Section III – Veterinarians involvement in zoonotic diseases prevention**		
**Please tick *(√)* or fill in the blank**		
18. Do you recommend the use of collars to prevent leishmaniosis?	Cats<br>☐ Yes<br>☐ No<br>☐ Only if the animals live outside the house	Dogs<br>☐ Yes<br>☐ No<br>☐ Only if the animals live outside the house
