# Supplementary material for: Veterinary perspectives on the urbanization of leishmaniosis in Morocco
Source: Parasit Vectors. 2024 Aug 19;17:348. doi: 10.1186/s13071-024-06411-5 (PMC11334585; doi:10.1186/s13071-024-06411-5)
Supplement: Supplementary file 3 — Additional file 3: Table S2. Characterization of the canine study group (n = 156): description of number of sampled dogs and their demographic, clinical and prophylactic history. [file 13071_2024_6411_MOESM3_ESM.docx]

**Additional file 3: Table S2**. Characterization of the canine study group (n = 156): description of number of sampled dogs and their demographic, clinical and prophylactic history.

| Variable/  category | Number of  animals (n) | Relative distribution (%) | |
| --- | --- | --- | --- |
| Sex | 156 | 100 |  |
| Male | 70 | 44.8 |  |
| Neutered | 18 | 25.7 |  |
| Intact | 52 | 74.3 |  |
| Female | 86 | 55.1 |  |
| Intact | 19 | 22.1 |  |
| Spayed | 67 | 77.9 |  |
| Age (years) | 156 | 100 |  |
| Puppy (< 1) | 2 | 1.3 |  |
| Young adult (1–3) | 8 | 5.1 |  |
| Adult (3–9) | 144 | 92.3 |  |
| Mature (9–12) | 0 | 0.0 |  |
| Senior (> 12yrs) | 2 | 1.3 |  |
| Breed | 156 | 100 |  |
| Pure breed | 13 | 8.3 |  |
| Cross breed | 43 | 27.6 |  |
| No recognized breed | 100 | 64.1 |  |
| Location | 156 | 100 |  |
| Rabat | 81 | 51.9 |  |
| Shelter | 52 | 33.3 |  |
| Owned | 29 | 15.6 |  |
| indoor/ outdoor | 12 | 41.4 |  |
| outdoor | 17 | 58.6 |  |
| Fez | 75 | 48.1 |  |
| Shelter | 46 | 29.5 |  |
| Owned | 29 | 18.6 |  |
| Indoor/outdoor | 26 | 89.7 |  |
| Outdoor | 3 | 10.3 |  |
| Total shelter | 98 | 62.8 |  |
| Total owned | 58 | 37.2 |  |
| Clinical evaluation | 156 | 100 |  |
| Apparently healthy | 102 | 65.4 |  |
| CanL suspect | 48 | 30.8 |  |
| Prophylatic events | |  |  |
| External parasites | |  |  |
| Owned | 56 | 100 |  |
| Owner does not know | 2 | 3.4 |  |
| According to manufacturer (Afoxolaner, Fipronil) | 19 | 33.9 |  |
| Overdue (Sarolaner, Afoxolaner, tablet) | 34 | 60.7 |  |
| Never | 1 |  |  |
| Shelter | 98 | 100 |  |
| Up to date | 0 | 0.0 |  |
| Overdue | 98 | 100 |  |
| Internal parasites |  |  |  |
| Owned | 54 | 100 |  |
| Unknown | 3 | 3.4 |  |
| 1x/month | 24 | 33.3 |  |
| 2x/year | 2 |  |  |
| 2x/year | 7 | 10.5 |  |
| 4x/year | 1 | 1.8 |  |
| Never | 1 | 1.8 |  |
| > 1 year ago | 26 | 45.6 |  |
| Shelter | 98 | 100 |  |
| Up to date | 0 | 0.0 |  |
| Overdue | 98 | 100 |  |
| Vaccines |  |  |  |
| Owned | 58 | 100 |  |
| Unknown | 1 | 1.7 |  |
| Up to date | 29 | 50.0 |  |
| Overdue | 28 | 48.3 |  |
| Never | 0 | 0.0 |  |
| Shelter | 98 | 100 |  |
| Up to date | 0 | 0.0 |  |
| Overdue | 98 | 100 |  |
| Total | 156 | 100 |  |
