# Supplementary material for: Veterinary perspectives on the urbanization of leishmaniosis in Morocco
Source: Parasit Vectors. 2024 Aug 19;17:348. doi: 10.1186/s13071-024-06411-5 (PMC11334585; doi:10.1186/s13071-024-06411-5)
Supplement: Supplementary file 4 — Additional file 4: Table S3. Characterization of the feline study group (n = 33): description of number of sampled cats and their demographic, clinical and prophylactic history. [file 13071_2024_6411_MOESM4_ESM.docx]

**Additional file 4: Table S3**. Characterization of the feline study group (n=33): description of number of sampled cats and their demographic, clinical and prophylatic history

| Variable/  category | Number of  animals (n) | Relative  distribution (%) |
| --- | --- | --- |
| Sex | 31 | 93.9 |
| Male | 8 | 25.8 |
| Neutered | 7 | 87.5 |
| Intact | 1 | 12.5 |
| Female | 23 | 74.2 |
| Spayed | 0 | 0.0 |
| Intact | 23 | 100.0 |
| Age | 31 | 93.9 |
| Kitten (< 1) | 0 | 0.0 |
| Young adult (1 - 3) | 2 | 6.5 |
| Adult (3 - 9) | 29 | 93.5 |
| Mature (9 - 12) | 0 | 0.0 |
| Senior ( > 12) | 0 | 0.0 |
| Pure breed | 0 | 0.0 |
| Cross breed | 0 | 0.0 |
| Domestic short hair | 31 | 93.9 |
| Habitat | 33 | 100.0 |
| Stray | 27 | 81.8 |
| Owned | 6 | 18.2 |
| Indoor | 5 | 83.3 |
| Outdoor | 1 | 16.7 |
| Clinical evaluation | 31 | 93.9 |
| Apparently healthy | 20 | 64.5 |
| FeL suspect | 3 | 9.6 |
| Total | 33 | 100 |
