# Supplementary material for: Veterinary perspectives on the urbanization of leishmaniosis in Morocco
Source: Parasit Vectors. 2024 Aug 19;17:348. doi: 10.1186/s13071-024-06411-5 (PMC11334585; doi:10.1186/s13071-024-06411-5)
Supplement: Supplementary file 5 — Additional file 5: Table S4. Clinical manifestations in 48 dogs suspected of canine leishmaniosis (CanL). [file 13071_2024_6411_MOESM5_ESM.docx]

**Additional file 5: Table S4.** Clinical manifestations in 48 dogs suspect of canine leishmaniosis (CanL)

| Clinical manifestations  of CanL | No. of dogs | Percentage (%) of clinical manifestation in CanL suspect dogs |
| --- | --- | --- |
| Skin lesions |  |  |
| Generalized alopecia | 15 | 31.25 |
| Seborrhea | 3 | 2.1 |
| Crusts | 11 | 20,4 |
| Pyoderma | 1 | 2.1 |
| Onychogryphosis | 34 | 70.8 |
| Pinna lesions/ crusts | 2 | 4.2 |
| Generalized hyperkeratosis | 1 | 2.1 |
| Hyperkeratosis of the elbows | 1 | 2.1 |
| Skin nodules | 1 | 2.1 |
| Hyperpigmentation | 1 | 2.1 |
| Ocular lesions |  |  |
| Conjunctivitis | 3 | 2.1 |
| Periocular alopecia | 1 | 2.1 |
| Mucocutaneous lesions |  |  |
| Nasal planum hyperkeratosis | 5 | 10.41 |
| Systemic disease |  |  |
| Kidney disease | 1 | 2.1 |
| Lymphadenopathy | 26 | 54.2 |
| Anemia (pale mucous membranes) | 3 | 6.25 |
| Cachexia | 6 | 12.5 |
| Weight loss | 10 | 20.8 |
|  |  |  |
