# Supplementary material for: Veterinary perspectives on the urbanization of leishmaniosis in Morocco
Source: Parasit Vectors. 2024 Aug 19;17:348. doi: 10.1186/s13071-024-06411-5 (PMC11334585; doi:10.1186/s13071-024-06411-5)
Supplement: Supplementary file 6 — Additional file 6: Table S5. Clinical manifestations in three cats suspected of feline leishmaniosis (FeL). [file 13071_2024_6411_MOESM6_ESM.docx]

**Additional file 6: Table S5.** Clinical manifestations in 3 cats suspect of feline leishmaniosis (FeL)

| Clinical manifestations  of FeL | No. of cats | Percentage (%) of clinical manifestation in FeL suspect cats |
| --- | --- | --- |
| Skin lesions |  |  |
| Generalized alopecia | 3 | 100 |
| Seborrhea | 2 | 66.7 |
| Crusts | 2 | 66.7 |
| Generalized hyperkeratosis | 1 | 33.3 |
| Ocular lesions |  |  |
| Conjunctivitis | 1 | 33.3 |
| Non-specific signs |  |  |
| Cachexia | 2 | 66.7 |
