# Supplementary material for: Veterinary perspectives on the urbanization of leishmaniosis in Morocco
Source: Parasit Vectors. 2024 Aug 19;17:348. doi: 10.1186/s13071-024-06411-5 (PMC11334585; doi:10.1186/s13071-024-06411-5)
Supplement: Supplementary file 7 — Additional file 7: Table S6. Prevalence of Leishmania spp. in 155 dogs from Rabat and Fez, Morocco, as determined by polymerase chain reaction (PCR), according to categories of independent variables sex, breed, housing, city and presence of clinical manifestations compatible with canine leishmaniosis (CanL). [file 13071_2024_6411_MOESM7_ESM.docx]

**Additional file 7: Table S6.** Prevalence of *Leishmania* spp. in 155 dogs from Rabat and Fez, Morocco, as determined by polymerase chain reaction (PCR), according to categories of independent variables sex, breed, housing, city and presence of clinical manifestations compatible with canine leishmaniosis (CanL).

| Variable/  category | No. (%) of dogs tested | Percentage (n) of PCR-positive | 95% CI (%)^a^ |
| --- | --- | --- | --- |
| Sex | 153 (98.7) | *p* = 0.856 |  |
| Female | 87 (56.1) | 20.7 (18) | 12.8−30.7 |
| Male | 66 (42.6) | 18.2 (12) | 9.8−29.6 |
| Breed | 153 (98.7) | *p* = 0.063 |  |
| Mongrel | 115 (74.2) | 23.5 (27) | 16.1−32.3 |
| Defined/crossbred | 38 (24.5) | 7.9 (3) | 1.7−21.4 |
| Housing | 155 (100) | *p* = 0.056 |  |
| Shelter | 98 (63.2) | 24.5 (24) | 16.4−34.2 |
| Domestic | 57 (36.0) | 10.5 (6) | 4.0−21.5 |
| City | 155 (100) | *p* = 1.0 |  |
| Rabat | 80 (51.6) | 18.8 (15) | 10.9−29.0 |
| Fez | 75 (48.4) | 20.0 (15) | 11.7−30.8 |
| CanL clinical manifestations | 155 (100) | *p* = 0.028^c^ |  |
| Absent | 106 (68.4) | 14.2 (15) | 8.1−22.3 |
| Present^b^ | 49 (31.6) | 30.6 (15) | 18.3−45.4 |
| Total | 155 (100) | 19.4 (30) | 13.5−25.5 |

^a^ 95% confidence interval; ^b^ Two or more clinical manifestations compatible with CanL, comprising: lymphadenopathy, skin disorders, onychogryphosis, ophthalmic disorders, cachexia, fever, anemia and kidney disease; ^c^ Statistically significant difference (*p* < 0.05).
