# Supplementary material for: Veterinary perspectives on the urbanization of leishmaniosis in Morocco
Source: Parasit Vectors. 2024 Aug 19;17:348. doi: 10.1186/s13071-024-06411-5 (PMC11334585; doi:10.1186/s13071-024-06411-5)
Supplement: Supplementary file 8 — Additional file 8: Table S7. Partial kinetoplast DNA (kDNA) sequences identified in the three kDNA-positive cats from the study. [file 13071_2024_6411_MOESM8_ESM.docx]

| **ID** | **Partial kDNA sequence** |
| --- | --- |
| MOR.FEL.06 | TGGGTGCAGAATCCCGTTCAAAAATCGGCCAAAATGCCAAAAATCGGCTCCGGGGCGGGAAACTGGGGGTTGGTGTGGGGGTTGCCAGGTG |
| MOR.FEL.11 | TGGGTGCAGAATCCCGTTCAAAATTGGCCAAAATGCCATATTTCGGCTCCGGGGCGGGAAACTGGGGGTTGGTGTAAAATAAGCCAGGTGGG |
| MOR.FEL.23 | TGCAGAAATCCCGTTCAAAATTGCCCAAAAATGCCAAATTTCGGCTCCGGGGCGGGAAACTGGGGGTTGGTGTAAAATAGGCCAGGTGGG |

**Additional file 8: Table S7.** Partial kinetoplast DNA (kDNA) sequences identified in the three kDNA-positive cats from the study.
