# Supplementary material for: Veterinary perspectives on the urbanization of leishmaniosis in Morocco
Source: Parasit Vectors. 2024 Aug 19;17:348. doi: 10.1186/s13071-024-06411-5 (PMC11334585; doi:10.1186/s13071-024-06411-5)
Supplement: Supplementary file 9 — Additional file 9: Table S8. Demographic characteristics of Moroccan veterinarians participating in the questionnaire. [file 13071_2024_6411_MOESM9_ESM.docx]

**Additional file 9: Table S8.** Demographic characteristics of Moroccan veterinarians participating in the questionnaire. Analysis based on the data obatined from 50 fully completed questionnaires.

| Variable/  categories | Number of participants | Relative  distribution (%) |
| --- | --- | --- |
| Gender |  |  |
| Female | 30 | 60 |
| Male | 20 | 40 |
| Age | | |
| 24 - 28 | 35 | 70 |
| 29 – 33 | 9 | 18 |
| 34 - 39 | 2 | 4 |
| 40 – 45 | 1 | 2 |
| 45 – 50 | 1 | 2 |
| > 50 - 65 | 2 | 4 |
| Veterinary Studies |  |  |
| IAV Hassan II | 50 | 100 |
| Years in practice | | |
| Less than 2 yrs | 28 | 56 |
| Between 2 and 5 yrs | 12 | 24 |
| Between 5 and 15 yrs | 6 | 12 |
| More than 15 yrs | 4 | 8 |
| Type of practice | | |
| Small Animal Practice | 31 | 62 |
| Large Animal Practice | 3 | 6 |
| Mixed Practice | 13 | 26 |
| Reference/ University Hospital | 3 | 6 |
| Location | | |
| Urban | 42 | 84 |
| Rural | 8 | 16 |
| State province and cities |  |  |
| Casablanca-Settat | 32,7 | 16 |
| Casablanca, Mohammedia, Berrechid, Bir Jdid, Sidi Bennour |  |  |
| Rabat-Salé-Kénitra | 36,7 | 18 |
| Rabat, Salé, Temara, Sidi Allal El Bahraoui, Kenitra, Tifelt, Sidi Slimane |  |  |
| Fès-Meknès | 8,2 | 4 |
| Fez, Meknès, Taounate |  |  |
| Béni Mellal-Khénifra | 2,0 | 1 |
| Beni Mellal |  |  |
| Tanger-Tetouan-Al Hoceima | 4,1 | 2 |
| Tangier, Ayacha |  |  |
| Oriental | 2,0 | 1 |
| Oujda, Nador |  |  |
| Marrakesh-Safi | 2,0 | 1 |
| Echemmaia, Marrakesh, Sid L Mokhtar |  |  |
| Laâyoune-Boujdour-Sakia El Hamra | 4,1 | 2 |
| Laayoune |  |  |
