# Supplementary material for: Veterinary perspectives on the urbanization of leishmaniosis in Morocco
Source: Parasit Vectors. 2024 Aug 19;17:348. doi: 10.1186/s13071-024-06411-5 (PMC11334585; doi:10.1186/s13071-024-06411-5)
Supplement: Supplementary file 10 — Additional file 10: Table S9. Moroccan veterinarians’ responses to questions related to the epidemiology of leishmaniosis in Morocco (n = 50). [file 13071_2024_6411_MOESM10_ESM.docx]

**Additional file 10: Table S9**. Moroccan veterinarians responses to questions related to the epidemiology of leishmaniosis in Morocco (n = 50).

| Variable/categories | Number  of  replies | Relative  distribution (%) |
| --- | --- | --- |
| Knowledge of leishmaniosis zoonotic potential | | |
| Recognize leishamaniosis as a zoonotic disease | 37 | 74 |
| Not awere of transmission mode of leishmaniosis | 13 | 26 |
| Knowledge on vectorial transmission of *Leishmania* parasites | | |
| Awere | 49 | 98 |
| Not awere | 1 | 2 |
| Identification of causative agent of canine and feline leishmaniosis | | |
| Identification of *Leishmania infantum* | 38 | 76 |
| Identification of *Leishmania major* | 4 | 8 |
| Identification of *Leishmania tropica* | 0 | 0 |
| Doesn´t know | 8 | 16 |
| Identification of *L. infantum* primary reservoir hosts | | |
| Dogs | 27 | 54 |
| Humans | 1 | 2 |
| Cats | 0 | 0 |
| Desert roedents (genus Meriones) | 4 | 8 |
| Dogs, cats and humans | 1 | 2 |
| Dogs and humans | 2 | 4 |
| Dogs and cats | 3 | 6 |
| Dogs and desert roedents (genus Meriones) | 3 | 6 |
| Mouse, cat and dog | 1 | 2 |
| Mouse and dog | 1 | 2 |
| Doesn´t know | 7 | 14 |
| Identification of *L. major* primary reservoir hosts | | |
| Dogs | 4 | 8 |
| Humans | 6 | 12 |
| Cats | 0 | 0 |
| Desert roedents (genus Meriones) | 12 | 24 |
| Dogs, cats and humans | 0 | 0 |
| Dogs and humans | 0 | 0 |
| Dogs and cats | 1 | 2 |
| Dogs and desert roednts | 0 | 0 |
| Desert roedents | 4 | 8 |
| Mouse | 5 | 10 |
| Mouse, desert roedents and humans | 1 | 2 |
| Dog and desert roedents | 1 | 2 |
| Doesn´t know | 16 | 32 |
| Identification of *L. tropica* primary reservoir hosts | | |
| Dogs | 5 | 10 |
| Humans | 10 | 20 |
| Cats | 0 | 0 |
| Desert roedents | 7 | 14 |
| Dogs, cats and humans | 0 | 0 |
| Dogs and humans | 1 | 2 |
| Dogs and cats | 0 | 0 |
| Dogs and desert roedents | 0 | 0 |
| Mouse | 2 | 4 |
| Mouse, desert roedent | 0 | 0 |
| Mouse, desert roedents and humans | 0 | 0 |
| Dog and desert roedents | 0 | 0 |
| Dog, mouse, human | 2 | 4 |
| Desert roedent, human | 1 | 2 |
| Doesn´t know | 22 | 44 |
| Identification of *Leishmania* transmission vectors | | |
| Phlebotomine sand flies | 45 | 90 |
| Other arthropode vectors (e.g mosquitos, ticks, fleas) | 4 | 8 |
| Doesn´t know | 1 | 2 |
| Identification of *L. infantum* primary reservoir hosts | | |
| Dogs | 27 | 54 |
| Humans | 1 | 2 |
| Cats | 0 | 0 |
| Desert roedents | 4 | 8 |
| Dogs, cats and humans | 1 | 2 |
| Dogs and humans | 2 | 4 |
| Dogs and cats | 3 | 6 |
| Dogs and desert roedents | 3 | 6 |
| Doesn´t know | 7 | 14 |
| Mouse, cat and dog | 1 | 2 |
| Mouse and dog | 1 | 2 |
| Identification of *L. major* primary reservoir hosts | | |
| Dogs | 4 | 8 |
| Humans | 6 | 12 |
| Cats | 0 | 0 |
| Desert roedents | 12 | 24 |
| Dogs, cats and humans | 0 | 0 |
| Dogs and humans | 0 | 0 |
| Dogs and cats | 1 | 2 |
| Dogs and desert roedents | 0 | 0 |
| Doesn´t know | 16 | 32 |
| Mouse and desert roednets | 4 | 8 |
| Mouse | 5 | 10 |
| Mouse, desert roedents and humans | 1 | 2 |
| Dog and desert roedents | 1 | 2 |
| Identification of *L. tropica* primary reservoir hosts | | |
| Dogs | 4 | 8 |
| Humans | 6 | 12 |
| Cats | 0 | 0 |
| Desert roedents (genus Meriones) | 12 | 24 |
| Dogs, cats and humans | 0 | 0 |
| Dogs and humans | 0 | 0 |
| Dogs and cats | 1 | 2 |
| Dogs and desert roedents | 0 | 0 |
| Doesn´t know | 16 | 32 |
| Mouse and desert roednets | 4 | 8 |
| Mouse | 5 | 10 |
| Mouse, desert roedents and humans | 1 | 2 |
| Dog and desert roedents | 1 | 2 |
| Dog, mouse, human |  |  |
| Desert roedents and human |  |  |
| Previous diagnosis of animals leishmaniosis | | |
| Yes | 37 | 74 |
| Canine leishmaniosis | 36 | 97 |
| Other species | 1 | 3 |
| No | 13 | 26 |
