# Supplementary material for: Veterinary perspectives on the urbanization of leishmaniosis in Morocco
Source: Parasit Vectors. 2024 Aug 19;17:348. doi: 10.1186/s13071-024-06411-5 (PMC11334585; doi:10.1186/s13071-024-06411-5)
Supplement: Supplementary file 11 — Additional file 11: Table S10. Moroccan veterinarians’ responses to questions related to clinical management of leishmaniosis (n = 50). [file 13071_2024_6411_MOESM11_ESM.docx]

**Additional file 11: Table S10.** Moroccan veterinarians responses to questions related to clinical management of leishmaniosis (n = 50).

| Variable/  categories | Number of replies | Relative  distribution (%) |
| --- | --- | --- |
| Experience on previous diagnosis of animals leishmaniosis | 50 | 100 |
| Yes | 37 | 74 |
| No | 13 | 26 |
| Previous diagnosed of animal leishmaniosis, according to species | 50 | 100 |
| Canine | 36 | 72 |
| Feline | 0 | 0 |
| Horses | 0 | 0 |
| Donkeys | 1 | 2 |
| Never diagnoses leishmaniosis in an animal | 13 | 26 |
| Identification of clinical manifestations of leishmaniosis | 50 | 100 |
| No answer | 15 | 41 |
| Identified 1 clinical sign | 2 | 5 |
| Identified 2 clinical signs | 3 | 8 |
| Identified 3 or more clinical signs | 35 | 95 |
| Treatment options prescribed for animal leishmaniosis | 37 | 100 |
| Prescription of leishmanicidals/leishmaniostatitcs alone | | |
| Alopurinol | 5 | 13.5 |
| Meglumine antimoniate | 2 | 5.4 |
| Miltefosine | 1 | 2.7 |
| Alopurinol + Meglumine antimoniate | 2 | 5.4 |
| Alopurinol + Miltefosine | 1 | 2.7 |
| Alopurinol + domperidone | 1 | 2.7 |
| Prescription of leishmanicidal/ leishmaniostatic drugs in combination with antibiotics | | |
| Alopurinol + Meglumine antimoniate + doxycycline | 1 | 2.7 |
| Alopurinol + Doxycycline | 2 | 5.4 |
| Alopurinol + Marbofloxacin | 1 | 2.7 |
| Meglumine antimoniate + Doxycycline | 2 | 5.4 |
| Meglumine antimoniate + Marbofloxacin | 2 | 5.4 |
| Prescription of leishmanicidals/leishmaniostatitcs in combination with other parasiticidal drugs | | |
| Meglumine antimoniate + Imidocarb dipropionate | 1 | 2.7 |
| Alopurinol + Ivermectin | 1 | 2.7 |
| Prescription of leishmanicidals/leishmaniostatitcs in combination with other antimicrobials | | |
| Alopurinol + Marbofloxacin + Imidocarb dipropionate | 1 | 2.7 |
| Alopurinol + Marbofloxacin + Meglumine antimoniate | 5 | 13.5 |
| Other described therapeutics | | |
| Ivermectin + Doxycycline | 3 | 8.1 |
| Marbofloxacine + Doxycycline | 1 | 2.7 |
| Doxycycline + Meloxicam | 1 | 2.7 |
| Enrofloxacin + Marbofloxacine + Doxycycline | 1 | 2.7 |
| Doxycycline | 2 | 5.4 |
| Prophylatic recommendations for canine leishmaniosis | 50 | 100 |
| Usually recommend use of collars to prevent CanL | 12 | 24 |
| Don´t recommend use of collars to prevent CanL | 32 | 64 |
| Only recommend to dogs living outdoors | 6 | 12 |
| Prophylatic recommendations for feline leishmaniosis | 50 | 100 |
| Usually recommend use of collars to prevent FeL | 3 | 6 |
| Don´t recommend use of collars to prevent FeL | 41 | 82 |
| Only recommend to cats living outdoors | 6 | 12 |
| Knowledge of international guidelines for animal leishmaniosis (i.e. LeishVet, ESCCAP or CLWG) | 50 | 100 |
| Yes | 7 | 14 |
| No | 43 | 86 |
